# Effects of exercise prehabilitation before anterior cruciate ligament reconstruction on functional outcomes during pre- and postoperative rehabilitation — protocol for a single-blinded randomised controlled trial

**DOI:** 10.1186/s13063-023-07776-1

**Published:** 2023-11-24

**Authors:** Rebecca Abel, Daniel Niederer, Christoph Offerhaus, Sven Shafizadeh, Alexander Glowa, Ingo Froböse, Christiane Wilke

**Affiliations:** 1 German Sport University Cologne, Institut für Bewegungstherapie und bewegungsorientierte Prävention und Rehabilitation – Abt. 1., Am Sportpark Müngersdorf 6, 50933 Cologne, Germany; 2 Department of Sports Medicine and Performance Physiology, Goethe University Frankfurt, Sophienstr. 1-3, 60487 Frankfurt am Main, Germany; 3 Department of Orthopedic Surgery and Sports Traumatology, Sana Medical Centre, Witten/Herdecke University, Aachener Str. 445-449, 50933 Cologne, Germany; 4 PhysioSport PACE GmbH, Schanzenstraße 33, 51063 Cologne, Germany

**Keywords:** Knee, Prehabilitation, Preoperative training, ACL, RCT, Intervention

## Abstract

**Background:**

Although a benefit of preoperative training prior to anterior cruciate ligament (ACL) reconstruction is likely, there is no consensus on the optimal content (criteria-based programme), supervision (one-on-one guidance or self-administered training) and general setting of preoperative training after ACL injuries. The purpose of this trial is to investigate the efficacy of an individually adaptive, guided, structured and criteria-based preoperative rehabilitation programme in comparison to a non-guided and self-administered home training programme.

**Methods:**

The planned single-blinded randomised controlled trial study was approved by the ethics committee of the German Sport University on June 14, 2022 (ethics application no. 093/2022) and prospectively registered (DRKS-ID: DRKS00030312; date of registration: 26.09.2022). *N* = 114 participants between 16 and 60 years of age with a unilateral ACL rupture and scheduled ACL reconstruction with a hamstring or quadriceps tendon autograft will be randomly (block-randomisation, 1:1 allocation) and blinded assigned to one of two groups: intervention group (structured, criteria-based, guided prehabilitation training) and comparator group (non-guided, self-administered home training). After surgical reconstruction, patients of both groups participate in the same standard, functional measurement-guided, postoperative rehabilitation programme. Stepwise increasing the functional requirements of the assessments, all participants participate in testing at the day of anamnesis (t1), 1–7 days before surgical reconstruction (t2), day of surgical reconstruction (t3) and 30 (t4), 60 (t5), 90 (t6) and 180 (t7) days post-reconstruction.

The primary outcome is the overall self-reported knee condition, assessed by the sum score of all sub-scales of the Knee injury and Osteoarthritis Outcome Score (KOOS). Secondary outcomes include functional outcomes (range of motion, knee flexors and extensors and plantar flexors strength/torque, functional postural control, jumping ability), workability and return to sport (RTS) (psychological readiness, RTS success).

**Discussion:**

The planned study targets to fill a gap in the evidence regarding effective designs of prehabilitation training before surgical ACL reconstruction. Potential difficulties that could affect the conduct of the study are lack of treatment adherence of the patients and high dropout.

**Trial registration:**

German Register of Clinical Trials DRKS-ID: DRKS00030312. Registered on 26 September 2022.

**Supplementary Information:**

The online version contains supplementary material available at 10.1186/s13063-023-07776-1.

## Background

The rupture of the anterior cruciate ligament (ACL) is one of the most common and severe knee injuries [1–3]. Athletes are more endangered to rupture their ACL than inactive individuals [1–3]. In particular in such active patients, the surgical reconstruction of the ACL is the evidence-based rehabilitation and most common treatment after an ACL rupture [4, 5].

Ruptures of the ACL often lead to knee osteoarthritis and secondary instability in the knee joint [6]. Athletes returning to sports after ACL injury have a up to six-fold increased probability of recurrent injury up to 2 years postoperative compared to non-injured athletes [7]. They often perform worse and are characterised by shorter careers than non-injured counterparts [8]. These secondary and follow-up issues often occur after surgical reconstruction despite evidence-based treatments. As a consequence, the rehabilitation process after ACL injuries can still be improved in order to restore the function of the knee and to maintain physical activity and workability. The latter may vice versa lead to a decreased secondary issue or re-injury risk [9]. Clinical practice guidelines for anterior cruciate ligament rehabilitation recommend to include a prehabilitation phase between the injury and the reconstruction [5]. The suggested benefit of preoperative rehabilitation (prehabilitation) is based on the “better-in better-out” strategy. For example, the preoperative maintenance/improvement of conditional and coordinative performance, the improvement of perceived self-efficacy and the reduction of risk factors should enhance the pre- and postoperative functional outcomes and postoperative recovery [10]. The time between injury and reconstruction can vary from a few hours to several months. The later the surgery, the more time there is for preoperative preparation. Most patients are eligible for individually modified preoperative training after the inflammatory phase has ended (about 1 week after injury) [11].

But high-quality evidence investigating preoperative training is sparse. There is only low- to moderate-quality of evidence that prehabilitation approaches, in particular preoperative functional training, have a positive impact on preoperative and postoperative functional performance [12]. And there is only low-quality evidence that preoperative training has a positive effect on quadriceps strength, on performance in single-leg hop jumps 3 months postoperatively, and on self-perceived knee function and faster return to competition [12, 13]. Furthermore, there is no evidence-driven consensus regarding optimal content, supervision (one-on-one guidance or self-administered training) and general settings of preoperative training [12–14]. The available studies often lack an adequate comparator group, a sufficient follow-up time or important outcomes to provide evidence on the relevance of preoperative rehabilitation measures of potential secondary issues [12, 13].

## Methods

### Aims

The planned study focusses on optimal content (criteria-based programme), general setting of preoperative training and supervision (one-on-one guidance or self-administered training). With this randomised-controlled trial, our objective is to compare the effects of a guided, structured and criteria-based preoperative rehabilitation programme, which can be adapted to the individual performance level, to a non-guided and self-administered home-based training programme on functional and self-report outcomes and secondary issues (other knee function, daily living and return to sport). The KOOS sum score (Knee Injury and Osteoarthritis Outcome Score) is the primary outcome and assesses the self-perceived knee function [15, 16]. We hypothesise that the guided prehabilitation programme will be more effective than self-administered home training relating to its effect on the baseline to pre-reconstruction change score of the KOOS sum score.

We will investigate the effects of pre-operative training (guided vs. unguided) at different time points (pre-op, 30/60/90/180 days post-op). We expect that the guided prehabilitation programme will have a particularly positive short-term effect and that the difference between the two groups will decrease over the progress of rehabilitation.

### Study design

**Fig. 1:**
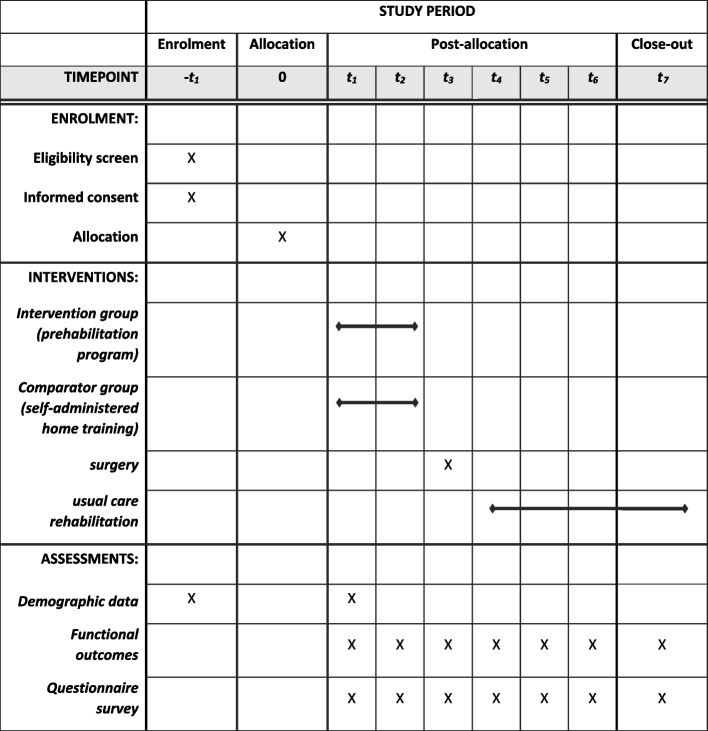
Schedule of enrolment, interventions and assessments (SPIRIT 2013, Standard Protocol Items: Recommendations for Interventional Trials) (t1 = first ambulant presentation in the hospital with confirming the diagnosis, selection of the operator and scheduling the operation, t2 = 1–7 days before surgery, t3 = surgery day, t4 = 30 days post OP, t5 = 60 days post OP, t6 = 90 days t7 = 180 days post OP)

This study is a monocentric, prospective randomised controlled single-blinded (patient-blind) trial. All patients receive an intervention, they do not know which group they are part of (intervention or comparator group). Ethical approval has been provided by the independent ethics committee of the German Sport University on June 14, 2022 (ethics application no. 093/2022). The study is prospectively registered (DRKS-ID: DRKS00030312; date of registration: 26.09.2022). The study was planned and performed in agreement with the Declaration of Helsinki (Version Fortaleza 2013). All adverse events will be reported. Informed consent is obtained by each participant prior to study enrolment. The protocol corresponds to the Standard Protocol Items of Recommendations for Interventional Trials (SPIRIT) guidelines (SPIRIT 2013 checklist) [17] (Fig. 1).

### Interventions

#### Prehabilitation programme (intervention group)

During the period between injury and surgical reconstruction, the participants of the intervention group perform a structured, criteria-based, guided training at PhysioSport PACE GmbH. The training programme will be adapted to the individual performance level and consists of two training sessions of 60 min per week. In addition, participants receive exercises once a week for self-administered training at home.

The prehabilitation programme is increased progressively and includes the following components [12, 18]

- Mobilisation of the knee joint, improvement of mobility
- Improvement of neuromuscular control, especially of the musculus quadriceps
- Improvement of postural stability and balance
- Restoration or increase of muscle strength (especially knee flexors and extensors, abductors, plantar flexors; local muscle strength endurance training, increase of muscle section, increase of neuromuscular strength).

**Fig. 2:**
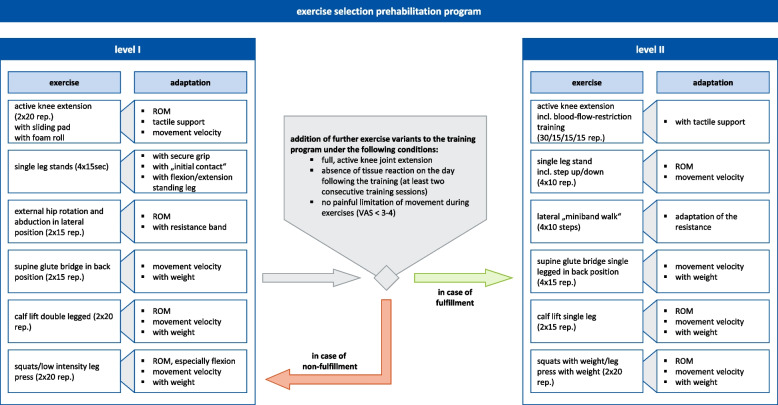
Exercise selection of the prehabilitation programme (level I and level II), recommended number of repetitions and adaptation possibilities of the exercises to lower/higher performance levels (ROM = range of motion, VAS = visual analogue scale, rep. = repetitions)

The choice of exercises is consistent with the exercises in previous studies [12]. In our planned trial, the prehabilitation programme is criteria-based and includes exercises at two levels (level I and level II). The exercise selection and progression are shown in Fig. 2.

At the beginning therapists at PhysioSport adapt exercises from level I to the individual performance level of the participants. If the participants have full, active knee joint extension, no tissue reaction on the following day of training (at least two consecutive training sessions) and no painful movement restrictions during the exercises (visual analogue scale (VAS) of 0–10 for subjective measurement of pain, VAS < 3–4), the exercise selection can be extended or replaced with exercises from level II by the therapist.

There is no time limit for achieving a level and differences in participants’ performance are to be expected. Therefore, it is conceivable that participants do not perform all the exercises of level II prior to surgical reconstruction and only perform the exercises from level I. The training programme aims to increase individual performance and not to achieve a defined performance level.

The therapists prospectively document the type, intensity, frequency and duration of all training sessions at PhysioSport, the self-administered training sessions performed at home and other treatments (e.g. physiotherapy) or unintended effects.

#### Comparator: self-administered home training

In clinical care, the patients usually do not receive any supervised, criteria-based, active therapy prior to surgical treatment. They are most often only asked to exercise on their own. The participants in the comparator group are also asked to exercise on their own and receive a brochure containing six exercises (active knee extension, single leg stands, external hip abduction, hip bridge in the back position, calf lift double legged, squats). These exercises pursue the same goals as the exercises in the guided prehabilitation programme. Participants find information in the brochure about the execution of the exercises and the number of repetitions. The brochure also contains information on the progression of the exercises. As they are all based on usual care exercises, the selection of the exercises and their progression are accompanied by a low risk of injury and follow the same principles as those in the intervention group. The treating physicians at the hospital recommend the participants of the comparator group to perform the exercises three times a week as self-administered, preoperative home training. The participants document independently all self-administered training sessions and other unintended effects. Patients will present this protocol to PhysioSport at preoperative testing. If participants have any questions, they can contact the study management. The self-administered training was chosen as the comparator to investigate the general setting and supervision (one-on-one guidance or self-administered training) of preoperative training after ACL injuries in this trial.

### Postoperative rehabilitation programme

After surgical treatment participants of both groups participate in a standard, guided, postoperative rehabilitation programme at PhysioSport. The rehabilitation programme initially focuses on postoperative follow-up treatment (wound and scar treatment, reduction of swelling, etc.). Subsequently, the goals already defined in the prehabilitation phase are pursued (mobilisation of the knee joint/ improvement of mobility, improvement of neuromuscular control especially of the m. quadriceps, improvement of postural stability/balance, restoration/increase of muscle strength). Monitoring of rehabilitation ends 180 days after surgery.

### Sample — inclusion criteria

Persons aged 16–60 years with unilateral and total primary ACL rupture (confirmed by magnetic resonance imaging) with indication for an arthroscopically assisted, anatomic ACL reconstruction using a hamstring or quadriceps tendon autograft who will be operated at Sana Medical Centre Cologne (inpatient or outpatient) are screened for inclusion. Participants can be included if the length of the period between injury and surgery is at least 3 and no longer than 13 weeks. Only participants who are able to give consent will be included. A parent or guardian must also sign the consent form for non-adult participants (16 or 17 years of age).

### Sample — exclusion criteria

Patients with concomitant injuries with an intrinsic indication for surgery that led to a change in the rehabilitation protocol and patients with prior operations or injuries to the knee joint (both to the injured leg and to the contralateral leg) in the last 5 years are excluded.

Comorbidities that represent a contraindication for an active training programme (e.g. severe cardiovascular diseases, neurological diseases …) as well as underlying rheumatic diseases and pregnancy are also exclusion criteria.

### Recruitment

Participants are recruited at Sana Medical Centre in Cologne. During anamnesis (systematic interviewing of patients and collection of health data), the treating physicians apply the inclusion and exclusion criteria to screen and subsequently recruit the patients for participation if they give their voluntary informed consent.

The voluntary informed consent form must be personally signed and dated by the participants. The participants receive written information about the study, about possible effects on the functionality of the knee joint (e.g. improvement of mobility and strength) and possible risks (e.g. aching muscles after training) associated with study participation. There is no anticipated harm and compensation for trial participation.

Study participants can withdraw their consent for study participation at any time without giving reasons and without affecting subsequent treatment.

### Study procedure

**Fig. 3:**
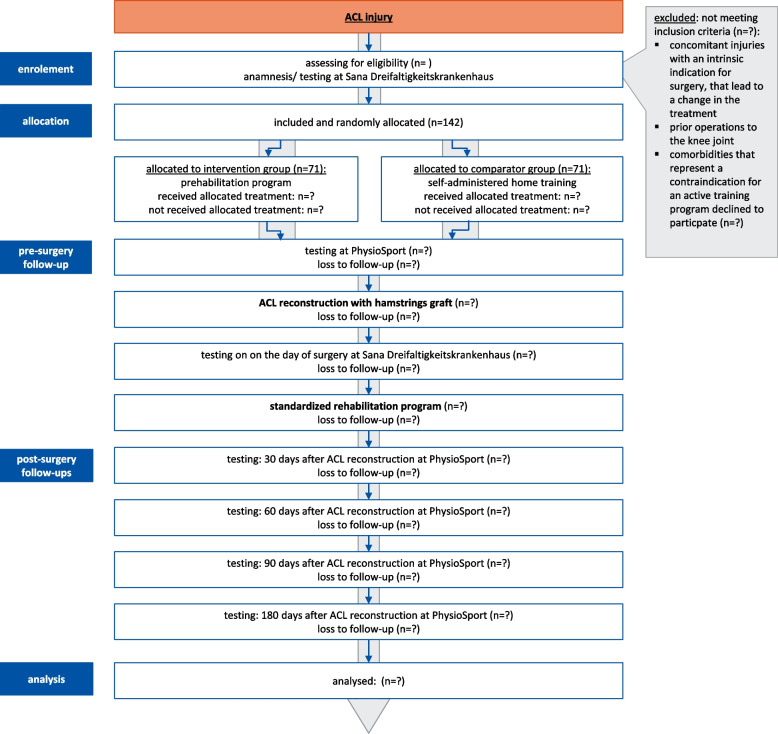
Study flow chart: study procedure (enrollment, allocation, pre-surgery follow-up, post-surgery follow-ups) (ACL = anterior cruciate ligament)

After ACL injury, anamnesis/testing at the hospital and randomised allocation, participants perform the prehabilitation programme (intervention group: guided prehabilitation programme, comparator group: self-administered home training) (Fig. 3). The training starts at the earliest possible date and lasts until reconstruction (duration at least 3 weeks). For organisational reasons (scheduling of appointments at the hospital) as well as for medical reasons (increased risk of arthrofibrosis during surgery in the inflammatory phase), the period between injury and surgery is at least 3 weeks for most patients at the hospital. The length of the period between injury and surgery as well as all medical decisions (timing of surgery, graft used, etc.) are made independently of the planned study.

Prior to surgical reconstruction (1–7 days before surgical reconstruction), the participants of both groups perform tests to assess the functionality of the knee joint guided by a therapist and complete selected questionnaires. Selected functional tests directed by a physician in the hospital and a questionnaire survey are also performed on the day of surgical reconstruction (postoperatively). There will be no special criteria for discontinuing or modifying allocated interventions. Patients will not be excluded from the study based on intention to treat. However, non-compliance will be monitored and reported.

While the prehabilitation programmes differ before surgery (guided training vs. home training), all participants participate in a standardised rehabilitation programme at PhysioSport after surgical reconstruction (Fig. 3).

### Organisation

Employees of the German Sport University (trial steering, monitoring and organisational support), Sana Medical Centre Cologne (recruitment of patients) and PhysioSport PACE GmbH (implementation of testing and training) are involved in the coordination. Those responsible are in close contact to each other. In addition, there is a meeting every month.

## Outcomes

**Fig. 4:**
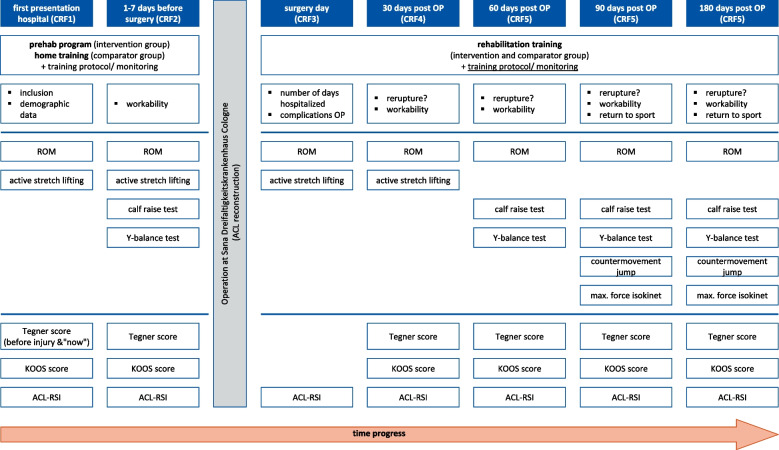
Participant timeline and testing-tools (CRF = case report file, OP = operation, ROM = range of motion, KOOS Score = Knee Injury and Osteoarthritis Outcome Score, ACL-RSI = anterior cruciate ligament—Return to Sport Injury Scale)

Functional parameters as well as self-reported knee function will be collected in both groups at seven defined measurement time points (anamnesis in the hospital, 1–7 days before ACL reconstruction, on the day of surgery, and 30, 60, 90, and 180 days postoperative) (Fig. 4). The primary time point is 1–7 days before ACL reconstruction.

### Primary outcome: self-reported knee function

The primary outcome of the study is the participants' self-perceived knee function, which is measured by the Knee Injury and Osteoarthritis Outcome Score (KOOS). The KOOS score is a measurement tool to assess participants' self-perceived knee function, especially after knee injuries such as ACL injuries [15, 19].

The KOOS score consists of 42 items in five subscales: pain, symptoms, function in daily living (ADL), function in sport and recreation (Sport/Rec) and knee-related quality of life (QOL). The mean of the subscale scores can be calculated and used to make an overall assessment of knee function [20]. The KOOS score has a high test–retest reliability. Overall, the minimum detectable change in patients with knee injuries is reported as 8–10 [16].

Since the KOOS score is used as the primary outcome in numerous randomised-controlled trials, there is a corresponding comparability of the results with other studies [19, 21]. Reference values of the KOOS score of patients after ACL reconstruction, of healthy subjects and active soccer players are also available and can be used for comparisons [22–25].

### Secondary outcomes

#### Functional outcomes

##### Range of motion

The active mobility of the knee joint (max. extension and flexion) is measured with a goniometer according to the neutral-zero method both preoperatively (following the anamnesis and 1–7 days before surgery) and postoperatively (on the day of surgery, 30 days and 60 days postoperative). The mobility of the knee joint is represented in a three-part code indicating the max. extension, the zero position as well as the max. flexion (e.g. 5°/0°/110°).

##### Neuromuscular control/activation

During the anamnesis, 1–7 days before the surgical reconstruction, on the day of the surgical reconstruction and 30 days after the surgical reconstruction, the participants are asked to perform an active extension of the leg while sitting (long sitting), i.e. to lift the extended leg from a mat (active stretch lifting = active lifting of the extended leg). The physician or therapist performing the test evaluates the performance as “not possible”, “possible with help” or “possible alone”.

##### Strength/torque (knee flexors and extensors, plantar flexors)

The strength of the knee flexors (hamstrings) and the knee extensors (m. quadriceps) is recorded 90 and 180 days postoperative using a strength test on the isokinet in a range of motion of 0°–90° (ROM 0°/0°/90°). For this purpose, maximum strength capacity is tested with 5 repetitions at 60°/s and strength endurance with 15 repetitions at 180°/s.

The strength of the plantar flexors (m. gastrocnemius) is tested with the “heel lift”. The participants stand upright with their legs extended and lift their heels off the floor to the maximum. Then one foot is lifted off the ground (single-leg stand) and the participants lift the heel off the ground maximally (plantar flexion). This test is performed both preoperatively (1–7 days before surgery) and postoperatively (after 60, 90 and 180 days). After 20 repetitions in full range of motion, the test is terminated. The therapist notes the number of repetitions the participant has completed in a side-by-side comparison (right/left) if the required number of 20 repetitions has not been reached.

##### Dynamic postural control

The dynamic postural control is measured by the Y-balance test. The Y-balance test is a functional and unilateral dynamic balance test in a single-leg stance and is performed by participants 1–7 days before surgery and 60, 90 and 180 days postoperative. It checks the range of motion of the playing leg in the three directions anterior, posteromedial and posterolateral (mobility of the joints: knee joints, hip joints, ankle joints) [26], the strength of the lower extremity [27], the proprioception [27], the dynamic balance [28] and the neuromuscular control, postural control, postural stability, as well as the trunk stability [27, 29]. The best values from both sides (left/right) and each direction (anterior/posteromedial/posterolateral) are selected from the valid trials and a total score is calculated. If the total score for one of the legs or for both legs is lower than 94%, there is an increased risk of injury [29]. The total scores (left/right) are used for further calculation of the LSI. The LSI (limb symmetry index) is used to determine whether a deficient lateral deviation is present [30].

##### Jumping ability

Counter Movement Jump is used to assess jumping abilities in a slow lengthening-shortening cycle. It is performed at 90 and 180 days postoperative and tests the explosive power, the maximum strength and the vertical bounce [31]. With the aid of a force plate, the flight time and the impulse during the jump are determined so that the jump height can be calculated. In addition, the force development is recorded in relation to the body weight as well as the percentage difference of the maximum force development in the bilateral comparison of the legs (affected vs. unaffected leg).

##### Workability

In order to record the workability, the number of days absent from work is determined preoperatively and postoperatively. In addition, it is determined when the participants state that they are fully able to work again.

#### Return to sport

##### Psychological readiness

The ACL-Return to Sport Injury Scale (ACL-RSI) is used to assess self-confidence, risk assessment, and emotions related to the ACL injury as well as complaints and problems caused by the knee joint injury. Participants answer a total of 12 questions on the 10-point scale from 0 to 100 1–7 days before surgery and 30, 60, 90, and 180 days postoperative [32]. A total score is determined from the results of the 12 questions answered (score achieved/number of questions). If the total score is below 51%, this indicates a lack of confidence in the knee joint. The decision to return to sport after an ACL injury is significantly related to response on the ACL-RSI scale [32].

##### Return to sport — success

The RTS success is characterised by achieving the pre-injury level of sports participation as defined by the same type, frequency, intensity, and quality of performance as before the injury.

The participant’s activity level is assessed at an initial presentation at the hospital (anamnesis), 1–7 days prior to surgery and 30, 60, 90 and 180 days postoperatively using the Tegner score (11-point scale). The Tegner score was developed specifically to assess the activity level of participants with ACL injuries and rates it on a scale from 1 (low activity level) to 10 (professional level) [33–35].

### Sample size determination

The sample size calculation is based on the sum score of the KOOS (primary outcome parameter). The difference in the KOOS score of the comparator and intervention group at the measurement pre-reconstruction will be compared. The minimal clinically important change (MIC) for the KOOS score is reported to be a change of 8–10 with respect to the subscale score in patients with knee injuries [36]. For performing a sample size calculation, usually, a standard deviation of SD = 15 is used for the KOOS score [16]. On this basis, an effect size of *d* = 0.533 was calculated using G*Power [37]. With a power of 80% and a one-sided alpha error level of 2.5%, data must be collected from *n* = 114 participants (equal number of participants in comparator and intervention group of *n* = 57 participants).

### Randomisation procedure

Participants will be randomly assigned to intervention or comparator group with a 1:1 allocation as per a computer-generated randomisation schedule using permuted blocks of random sizes. The block sizes will not be disclosed, to ensure concealment. All patients who give consent for participation and who fulfil the inclusion criteria will be randomised. Allocation concealment will be ensured, as the physician at the hospital will see the randomisation code not until the patient has been recruited into the trial. The physician will see the allocation after opening the opaque sealed envelopes.

### Data processing

The data collected (paper pencil, functional test results and questionnaires) are manually transferred to an electronic database (Excel, Microsoft Office Professional Plus 2019) and checked (range data check, outlier analysis) by the study management using R statistic (R version 4.2.3). Only employees involved in the study have access to the data. Collected data are treated confidentially, pseudonymized and evaluated exclusively scientifically. The data sets used and/or analysed in this study will be chaired open access in a publicly available data repository (Zenodo) at the end of the study period. A Data Monitoring Committee is not considered as this is a low-risk intervention.

### Statistical analysis

The two-sided alpha-error threshold will be set at 5% for all inference statistical analyses: all p-values below this value will be considered as statistically significant. All statistical analyses will be performed using R statistic (R version 4.2.3 or newer). We will follow the CHAMP statement when designing and reporting the statistical analysis [38].

Baseline data will be displayed as means and standard deviations, while the main outcome data will be displayed as means and 95% confidence intervals.

Following plausibility control (physiological range check, formal outlier analysis), the main analyses will be conducted based on the intention-to-treat principle. For that purpose, a multiple imputation, assuming missing completely at random and using chained equations in a fully conditional specification with 40 iterations to produce asymptotically unbiased estimations of the data, will be performed.

The group affiliation of the participants (intervention group: prehabilitation, comparator group: home training) will be defined as a between-subject factor. The main analyses are calculated as linear mixed models for repeated measurements. Main inference statistical analyses will be performed using each outcome’s change scores from baseline to each follow-up as the dependent variable. These analyses will be performed confirmatory. The time effects (repeated measures) will be modelled as random effects (and factors), and the other independent variables (group) and covariates (potential confounders and effect modifiers) as fixed effects. In the same model, the influence on the treatment effect by clinically important confounders and effect modifiers age, gender, and activity level will be calculated.

Patients, who do not have surgery, will not be included. If patients will decide after inclusion that they do not want surgery after all, the last testing will be done before the originally scheduled surgery date (intention-to-treat).

### Publication

The results of the study will be published in high-impact peer-reviewed journals to make them available to the orthopaedic and rehabilitation community. In addition, the results of the study will be presented and discussed at scientific conferences.

## Discussion

There is evidence on the relevance of prehabilitation prior to surgical ACL reconstruction to improve neuromuscular and self-assessed knee function, but only with low to moderate quality [12–14]. Further randomised-controlled studies of higher quality and methodology are needed to substantiate the importance of prehabilitation. The conditions of prehabilitation training must be concretized, i.e. the extent, setting, intensity, etc. of the prehabilitation training should be specified and psychological factors (e.g. psychological readiness for return to sport) should be recorded.

The planned study is a single centre and relatively small in size and likely will not be able to define the conditions of prehabilitation completely. Nevertheless, the planned study will help to fill a gap in the evidence regarding the setting of prehabilitation interventions before surgical ACL reconstruction (guided prehabilitation programme vs. self-administered home training).

The last testing in this study is done 180 days after surgery. A longer follow-up would possibly give more details on the potential participants who have still not returned to sports. In recently published studies the follow-up is done up to 3 months after surgery, we extended to 180 days. The follow-up period is therefore relatively long [12].

The conduct of the proposed study presents the following challenges that should be considered:

### Treatment adherence of the participants

In the intervention group, non-adherence to training appointments may reduce the success of prehabilitation training. In the comparator group, there is a risk of lack of motivation to perform home training independently without guidance. In addition, errors in performing the exercises cannot be corrected in the comparator group, since the training is self-administered and without guidance.

### Drop out

In preoperative training programmes that have already been implemented, participant’s compliance was high and the dropout rate was low [39–41]. Nevertheless, there is a risk of dropout in the planned study because the last testing takes place a long time after ACL reconstruction (180 days postoperative). The dropout of the study participation by the patients could distort the results. To keep the drop-out rate low, patients are regularly reminded of their testing dates, and after the testing, they receive their personal test results, which are helpful in assessing the rehabilitation process.

### Time between rupture and reconstruction

The time between injury and surgical treatment is individual and can vary considerably. In previous studies, the time between diagnosis of the ACL injury and surgical treatment was sometimes not reported [12]. To minimise the impact of the training period on outcomes, only participants with the opportunity to complete at least a 3-week training programme are considered eligible. Thus, there is still a risk of a positive influence on outcomes when training for more than 3 weeks.

### Recruiting participants

Only participants who can reach the selected training location (PhysioSport, PACE) for regular training will participate in the study. Thus, the recruitment of subjects might be difficult. It may be possible to extend the training locations to other locations/stores of PhysioSport.

## Trial status

Protocol version number 1

Date: 22/06/2023

Start of recruitment: 01/04/2023

Approximate date when recruitment will be completed: June 2024

### Supplementary Information

**Additional file 1.**

## Data Availability

The data sets used and/or analysed in this study will be chaired open access in a publicly available data repository.
